# Etiological study of esophageal squamous cell carcinoma in an endemic region: a population-based case control study in Huaian, China

**DOI:** 10.1186/1471-2407-6-287

**Published:** 2006-12-15

**Authors:** Zemin Wang, Lili Tang, Guiju Sun, Yuntian Tang, Yin Xie, Shaokang Wang, Xu Hu, Weimin Gao, Stephen B Cox, Jia-Sheng Wang

**Affiliations:** 1 Department of Environmental Toxicology and the Institute of Environmental and Human Health, Texas Tech University Lubbock, Texas, USA; 2 Southeast University, Nanjing, Jiangsu, P.R. China; 3 Center for Disease Control and Prevention, Chuzhou Branch, Huaian, Jiangsu, P.R. China

## Abstract

**Background:**

Continuous exposure to various environmental carcinogens and genetic polymorphisms of xenobiotic-metabolizing enzymes (XME) are associated with many types of human cancers, including esophageal squamous cell carcinoma (ESCC). Huaian, China, is one of the endemic regions of ESCC, but fewer studies have been done in characterizing the risk factors of ESCC in this area. The aims of this study is to evaluate the etiological roles of demographic parameters, environmental and food-borne carcinogens exposure, and XME polymorphisms in formation of ESCC, and to investigate possible gene-gene and gene-environment interactions associated with ESCC in Huaian, China.

**Methods:**

A population based case-control study was conducted in 107 ESCC newly diagnosed cases and 107 residency- age-, and sex-matched controls in 5 townships of Huaian. In addition to regular epidemiological and food frequency questionnaire analyses, genetic polymorphisms of phase I enzymes CYP1A1, CYP1B1, CYP2A6, and CYP2E1, and phase II enzymes GSTM1, GSTT1, GSTP1, and microsomal epoxide hydrolase (EPHX) were assessed from genomic DNA using PCR based techniques.

**Results:**

Consuming acrid food, fatty meat, moldy food, salted and pickled vegetables, eating fast, introverted personality, passive smoking, a family history of cancer, esophageal lesion, and infection with Helicobacter pylori were significant risk factors for ESCC (P < 0.05). Regular clean up of food storage utensils, green tea consumption, and alcohol abstinence were protective factors for ESCC (P < 0.01). The frequency of the *GSTT1 *null genotype was higher in cases (59.4%) compared to controls (47.2%) with an odds ratio (OR) of 1.68 and 95% confidence interval (CI) from 0.96 to 2.97 (P = 0.07), especially in males (OR = 2.78; 95% CI = 1.22–6.25; P = 0.01). No associations were found between polymorphisms of *CYP1A1*, *CYP1B1*, *CYP2A6*, *CYP2E1*, *GSTM1*, *GSTP1*, and *EPHX *and ESCC (P > 0.05).

**Conclusion:**

Our results demonstrated that dietary and environmental exposures, some demographic parameters and genetic polymorphism of *GSTT1 *may play important roles in the development of ESCC in Huaian area, China.

## Background

Esophageal cancer is the eighth most commonly occurring cancer and the sixth leading cause of cancer deaths in the world, with the majority of cases occurring in developing countries [1]. China, with ~250,000 cases diagnosed yearly, lies in the "esophageal cancer belt" [2], and contributes to about half of the world's cases [3]. The prevalence of esophageal cancer varies greatly across China, but there are two major endemic areas: the southern Taihang Mountain area (Linxian, Henan Province) and the northern Jiangsu area. In the northern Jiangsu area, the esophageal cancer incidence is over 80/100,000 which is six times greater than the national average rate (13/100,000). Many studies have been conducted in the southern Taihang Mountain area; however, despite very distinct geographic environments, fewer studies have been done in the northern Jiangsu area. Since esophageal squamous cell carcinoma (ESCC) represents more than 99% of esophageal cancer cases in China [4], this study focused only on ESCC.

Previous epidemiological studies have demonstrated that exposure to environmental carcinogens plays a significant role in the etiology of ESCC [5-7]. The recognized risk factors for ESCC include excessive use of tobacco, alcohol consumption, and the consumption of salt-pickled, salt-cured or moldy foods [8-10]. Other factors associated with increased risk of ESCC include vitamin and trace mineral deficiencies [11]. On the other hand, genetic polymorphisms may also play an important role in the development of ESCC and in determining individual susceptibility to carcinogens. Many studies have suggested that polymorphisms in xenobiotic-metabolizing enzymes (XME) including phase I enzymes such as CYP1A1, CYP1B1, CYP2A6 and CYP2E1, and phase II enzymes such as GSTM1, GSTT1, GSTP1, and microsomal epoxide hydrolase (EPHX) may confer different risks of susceptibility to cancer [12-20], including ESCC [21-23]. For example, genetic variations, including *CYP2E1 c1/c1 *allele, *GSTM1 *and *GSTT1 *non-null genotype and *EPHX *slow allele, were associated with increased risk of ESCC, while *CYP1A1 3' *polymorphism (variant allele) may be one of the protective factors for ESCC [22,23]. It is clear that many cancers including ESCC, are the result of complex interactions between inherited and environmental factors [24].

Although extensive studies have been carried out during the last two to three decades in the southern Taihang Mountain area [8,11,22,23], data are scarce from the northern Jiangsu area [21]. In the present study, we conducted a population based case-control study of ESCC in the Huaian area. In addition to regular epidemiological and food frequency questionnaire analyses, we investigated the polymorphisms of several phase I metabolic enzymes, including CYP1A1, CYP1B1, CYP2A6, and CYP2E1, and phase II metabolic enzymes, including GSTM1, GSTT1, GSTP1 and EPHX, which are believed to be involved in the metabolism of the environmental carcinogens for many types of cancers. The overall goal of this study is to evaluate the etiological roles of dietary carcinogen exposure and XEM polymorphisms, and to investigate possible gene-gene and gene-environment interactions associated with ESCC in this high risk area.

## Methods

### Study subjects

This population based matched case-control study consisted of 107 patients with ESCC and 107 healthy controls. All subjects were unrelated ethnic Han Chinese and residents in 5 townships of Chuzhou District, which were located at the north side of the General Irrigation Canal, Huaian City, Jiangsu Province, China. Based on a database at the malignant tumor registry of Huaian Center for Disease Control and Prevention (CDC), all cases were newly diagnosed with primary ESCC between 2002 and 2003. Cases were diagnosed at local hospitals and confirmed by endoscopy, X-ray or clinical histopathology. Control subjects were randomly selected healthy individuals confirmed by physical examination, clinical and biochemical analyses during the same period of case recruitment. Eligible controls included individuals without a history of cancer and were matched to cases on the basis of age (± 5 years), gender, and residency. Informed consent was obtained from each subject. The study protocol was approved by the Institutional Review Boards of the Texas Tech University, the Southeast University, and Huaian CDC for human subject protection. Sample collection, storage and shipment were complied with guidelines of both Chinese and US governments.

### Study procedures

A unified questionnaire, administered by trained personnel, was used to obtain study information. The questionnaire included demographic and residential characteristics, consisting of age, gender, height, weight, personal medical history, family cancer history, cigarette smoking, alcohol drinking, personality, dietary behaviors, green tea consumption, and exposure to pesticides and water-borne toxicants. Data on cigarette smoking included age started and ceased, number of cigarettes per day and duration. Participants were defined as current smokers if they smoked at least one cigarette per day for at least six months consecutively before the diagnosis for cases or to the date the controls were interviewed. Alcohol history included general information such as age started and ceased, as well as specific questions about the types of alcohol consumed, such as beer, yellow rice wine, red wine and liquor. Drinking status was defined as drinking at least once per day for at least six months continuously. The personality was classified into 3 categories including optimistic (or extrovert), relative optimistic (intermediate extrovert), and introvert. Extrovert was defined as sociable, active, assertive, energetic, animated, enthusiastic outgoing, impulsive, emotionally expressive, talkative, act before thinking, and broad experience. Introvert was defined as shy, unsociable, often appear reserved, quiet and thoughtful, usually do not have many friends, and have difficulties in making new contacts, like concentration and quiet. Esophageal lesion in our study was defined as including one or more of the following clinically diagnosed esophagus diseases or changes: 1) chronic esophagitis; 2) reflux esophagitis; 3) esophageal epithelium regeneration; 4) esophageal epithelium metaplasia; 5) esophageal polyps; and 6) esophageal erosion. All subjects were asked to fill out a quantitative food frequency questionnaire on 8 categories including 1) staple diet; 2) fried food; 3) pickled and salty food; 4) animal meat, egg and milk; 5) bean and bean product; 6) vegetables; 7) fresh fruits; and 8) nuts and dried fruits. A total of 120 food items were included in these 8 categories. These were considered representative of the usual diets of local residents.

In addition to epidemiological and food frequency questionnaires, 7 mL blood samples were collected from each subject, and separated into white blood cell (WBC) and plasma. Serological markers of Hepatitis B virus and Helicobacter pylori (HP) infection were detected. All samples were stored at -80°C and then shipped to the Institute of Environmental and Human Health, Texas Tech University for further analysis.

### DNA extraction and polymorphism analysis

Genomic DNA was extracted from WBC using the Qiagen QIAamp DNA Blood Mini Kit (QIAGEN Inc. Valencia, CA, USA) according to the procedure provided by the manufacture. Ten polymorphic alleles in 8 genes were investigated using PCR based techniques (Table 1). The *CYP1A1 *3'-UTR, *CYP1A1 *exon7, *CYP1B1, CYP2E1, GSTP1, EPHX113 and EPHX1 *genotypes were analyzed using PCR-RFLP methods as described previously [22,25-29]. A two step PCR was used for detecting the *CYP2A6 *deletion allele [30]. A modified multiplex PCR method was used for genotyping *GSTM1*, *GSTT1 *[23]. Briefly, the PCR was performed in a 25 μL reaction mixture containing 50 ng genomic DNA template, 1.5 mM MgCl_2_, 0.3 mM of each deoxynucleotide triphosphate (dNTPs) (MBI Fermentas Inc., Hanover, MD, USA), 0.8 μM (20 pmol) of each primer, 2.5 μL 10X PCR buffer and 1.25 Units Platinum Taq polymerase (Invitrogen, Carlsbad, CA, USA). A programmable thermocycler (PTC-200, MJ Research, Inc., MA, USA) was used. In each reaction, amplification of the *beta-globin *gene was used as an internal standard. The amplification protocol was set up for an initial denaturation of 2 min at 95°C, followed by a total of 30 cycles of 15 sec at 94°C, 30 sec at 61°C and 45 sec at 72°C. A final extension step was performed at 72°C for 5 min. The amplified products were visualized by electrophoresis on ethidium bromide-stained 2.0 % MetaPhor Agarose gel (Cambrex Bio Science Inc., Walkersville, MD, USA) in 1X TAE buffer.

For quality control, 10% of the samples were randomly selected and regenotyped for all of the selected genes, and the results were 100% concordant.

### Statistical analyses

Database on epidemiological and food intake frequency questionnaire data was established before analyses. Genotype frequencies among cases and controls were compared with values predicted by the Hardy-Weinberg equilibrium using χ^2 ^test. The association between individual variables including demographic characteristics, dietary and environmental factors, or genetic polymorphisms, and risk of ESCC was estimated by conditional logistic regression. Odds ratios (ORs) and their 95% confidence interval (CIs) were determined. The simultaneous effects of statistically significant variables on cancer risk were then examined using multivariable conditional logistic regression model. Interactions between genotypes and genotypes or between genotypes and environmental factors were also estimated by using conditional logistic regression. A p-value of ≤0.05 was considered statistically significant. The statistical analyses were performed with STATA 6.0.

## Results

### Subject characteristics

Demographic information of study subjects was summarized in Table 2. Mean age, gender distributions, family income, tobacco smoking, and alcohol drinking status did not differ between cases and controls (P > 0.05). Passive smoking and a family history of cancer were found to be risk factors for ESCC (P < 0.05). The percentages for optimistic, relative optimistic, and introvert personality were 49% (104/214), 41% (87/214), and 10% (23/214), respectively. Five percent (11/214) individuals had esophageal lesion and 34% (72/214) had HP infection. Individuals with an introverted personality, esophageal lesion, or previous HP infection were more likely to develop ESCC (P ≤ 0.05, Table 3).

### Associations of dietary behavioral factors and risk of ESCC

Among the 32 dietary behavioral factors in the epidemiological questionnaire, 12 variables showed statistically significant associations with ESCC (Table 3). Eating fast, eating fatty meat, pickled vegetables, acrid, salty, and moldy food were all risk factors of ESCC (P < 0.05); whereas, green tea consumption at 60~80°C, regular clean up of food storage utensils, and alcohol abstinence were protective factors of ESCC (P < 0.01).

### Associations of XME polymorphisms with risk of ESCC

**Figure 1:**
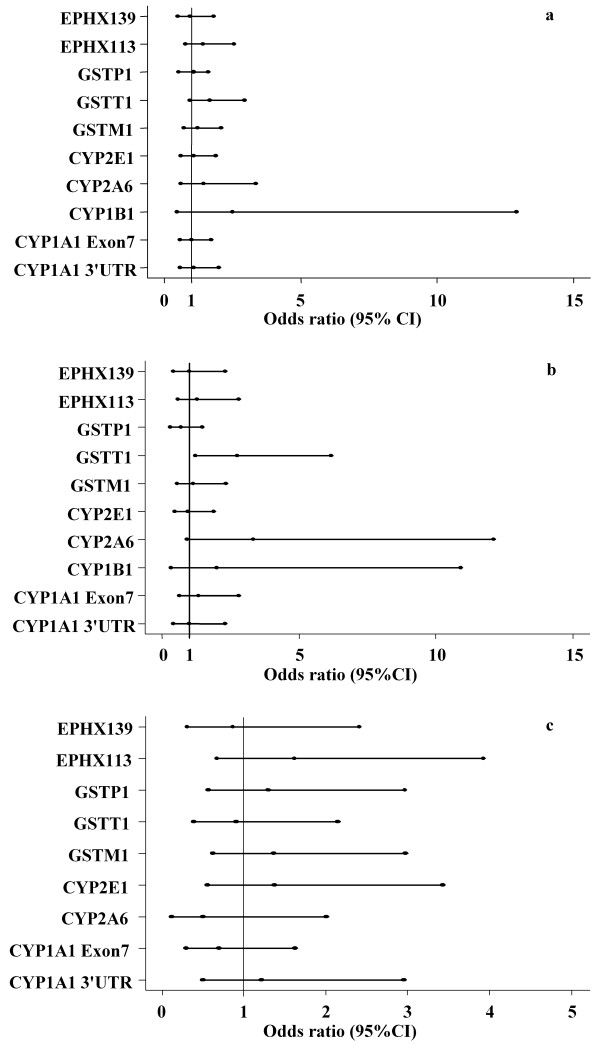
Xenobiotic metabolizing enzyme polymorphisms and risk of ESCC. Heterozygote and homozygote variant alleles were combined for each gene and wild type was used as reference. The middle dot of each line represents an odds ratios value, the left and the right dot of each line represent the lower and upper limits of 95% confidence interval, respectively. (a) Phase I and phase II enzyme polymorphisms and risk of ESCC among the study subjects. (b) Phase I and phase II enzyme polymorphisms and risk of ESCC in male subjects. (c) Phase I and phase II enzyme polymorphisms and risk of ESCC in female subjects.

Genotype frequencies did not deviate from the Hardy-Weinberg equilibrium among cases and controls (P > 0.05). Allele frequencies of phase I metabolic enzymes (including CYP1A13'-UTR, CYP1A1 exon7, CYP1B1, CYP2A6 (del), and CYP2E1) and phase II metabolic enzymes (including GSTM1, GSTP1, EPHX113, and EPHX139) were not significantly different between cases and controls (P > 0.05) (Figure 1a). In addition, no significant association was found between these genotypes and ESCC within either males or females (Figure 1b~1c). However, *GSTT1 *genotype was marginally associated with ESCC (OR = 1.68; 95% CI = 0.96–2.97; P = 0.07). This association was more significant in male patients (OR = 2.78; 95% CI = 1.22–6.25; P = 0.01) (Figure 1b).

### Multivariable conditional logistic analyses

When examined simultaneously, a family history of cancer, esophageal lesions, eating fast, and HP infection increased the risk of developing ESCC (Table 4), while regular clean up of food storage utensils and tea consumption were shown to be important protective factors with ORs of 0.11 (95% CI = 0.02–0.49; P < 0.01) and 0.13 (95% CI = 0.03–0.62; P = 0.01), respectively.

### Analyses of gene-gene and gene-environment interactions

Gene-gene and gene-environment interactions were examined by conditional logistic regression and no statistical significances were found (P > 0.05).

## Discussion

In the present study, introverted personality, passive smoking, a family history of cancer, esophageal lesion, HP infection, eating fast, fatty meat consumption, pickled vegetables intake, moldy food intake, favor to salty and acrid food were all associated with increased risk of ESCC. However, regular clean up of food storage utensils, alcohol abstinence lasted for more than half a year, and tea drinking were protective for ESCC. Among the factors associated with ESCC in this study, an introverted personality type, clean up of food storage utensils, and alcohol abstinence are first reported. Other major findings are consistent with previous studies conducted in China and elsewhere [31-33] including studies conducted in the Huaian area [34,35]. For instance, previous epidemiological surveys have shown that people in this area used to consume a lot of salted food and pickled vegetables. These foods are a rich source of nitrosamine compounds, and have been reported to be risk factors for ESCC [36]. Moldy foods are frequently contaminated by fungal toxins, and it has long been reported that mycotoxins, especially Fumonisin B_1 _(FB_1_), are associated with ESCC [37]. This may also be an explanation for our finding that maintaining clean storage utensils reduces the risk of ESCC. Damp and dirty environments favor the growth of mold and mildew, and regular clean up of the utensils may be a good habit to prevent food from developing mildew and molds. Eating fast, as well as frequently consuming acrid foods, which are common physical stimulations to the esophagus, most often will lead to the chronic esophagitis. This has been considered the earliest tissue perturbation in the progressive process leading to malignant transformation of the squamous epithelium of the esophagus [38].

We found that passive smoking is significantly associated with increased risk of ESCC. Passive smoking is a major risk factor for lung cancer, and emerging studies have shown that it may also be related to increased risk of breast cancer in women [39]. It has been suggested that passive smoking or environmental tobacco smoke (ETS), 85% of which consisted by sidestream smoke due to burning cigarette, was more risky than active smoking, and concentrations of some carcinogens are higher in sidestream than in mainstream smoke, which is exhaled by the smoker [40]. ETS has been classified as a Group A carcinogen which means it is known to cause cancer in humans [41]. The number of known carcinogens in ETS has been reported to be 69 in 2000 [42]. The role of active smoking has been examined by age started, smoking intensity, years smoked, cessation, type of tobacco, and type of cigarette. No association between active smoking and ESCC was found.

We also found that alcohol abstinence is a protective factor for ESCC. It is possible that genetic damage such as chromosome aberrations and micronuclei caused by alcohol are reversible by abstinence [43]. Although several epidemiological studies have showed that exposure to tobacco smoking and alcohol drinking are major risk factors for ESCC [44-46], we did not find any association between smoking and alcohol drinking and the risk of ESCC. It may be resulted from that men usually smoke and drink while women do not smoke or drink in this area.

Data from the present study revealed that HP infection was a risk factor for ESCC in the high risk population. However, results on HP infection and risk of ESCC have been inconsistent to date, an inverse relationship between HP infection and esophageal adenocarcinoma risk has been reported [47,48]. Furthermore, HP infection was protective against developing ESCC in a Taiwanese population [49]. Possible reasons for this discrepancy are not clear, and further investigation is needed.

Our study indicated that green tea is an important protective factor for ESCC, which is consistent with previous studies conducted in the same area [21] and in Shanghai, China [50]. Recent evidence suggests that the anti-oxidative and anti-inflammatory properties of green tea make it a promising agent for human cancer preventions [51]. In rats, green tea consumption prevents the esophageal carcinogenesis induced by N-nitrosomethylbenzylamine (NMBA), which is a potent carcinogen that is frequently found in moldy food and pickled vegetables [52]. A cohort study also showed an inverse relationship between urinary green tea polyphenols and risk of ESCC of men [53]. One possible mechanism may be that the green tea polyphenols induce epidermal growth factor receptor inhibition in esophageal cancer cells [54]. Taken together, these findings suggest that green tea might be used as a chemoprevention agent for human esophageal cancer.

Numerous studies have investigated the association between different XME polymorphisms and ESCC, but with conflicting results [18]. In the present study, a difference was found in the distribution of *GSTT1 *null genotypes between cases and controls; however, the difference only approached statistical significance. This difference was more striking in the male subjects, resulting in a 2.8-fold increase in risk of ESCC compared to male subjects who carried the *GSTT1 *non-null genotype. This suggests that the *GSTT1 *null genotype may contribute to the development of ESCC in this high risk population. Although this was different from previous studies on ESCC [22,23,55], it is biologically plausible because the presence of the *GSTT1 *null genotype leads to lower GST enzyme activity levels and, consequently, impaired detoxification of environmental or dietary carcinogens. In addition, *GSTT1 *null has been shown to play a role in esophageal carcinogenesis through a pathway of abnormalities in the *p53 *tumor suppressor gene, which is highly reported in ESCC [18]. Previous data showed that *CYP1A1 Val/Val *and *CYP2E1 c1/c1 *genotypes were related with increased risk of ESCC [22,56]. *EPHX *was also reported to be associated with high susceptibility of ESCC [23]. Nevertheless, we did not find significant association between *CYP1A1*, *CYP2E1*, *CYP2A6*, and *EPHX *and ESCC. The exact reasons for these inconsistent findings remain to be elucidated, but it is demonstrated that the variation in enzyme activity with ethnicity and gender may contribute to the differences in effects on the risk of cancers [57].

In order to find an overall risk for ESCC, variables with statistical significance in univariate conditional logistic regression were analyzed in a multivariable model. Esophageal lesion, eating fast, a family history of cancer, and HP infection were risk factors for ESCC (P < 0.05), while clean up of storage utensils and green tea consumption were protective factors for ESCC (P < 0.05). Personal character, fatty meat intake, pickled vegetables intake, and alcohol abstinence were not statistically significant in the multivariable model which might be due to their colinearity with other variables in the multivariable analysis. For instance, eating fast and pickled vegetables intake was highly correlated (P < 0.05, data not shown). Although, gene-gene and gene-environment interactions have been reported for ESCC [22,58], we did not find such interactions in our study, which might be related to the smaller sample size in our study.

## Conclusion

The present study indicated that fatty meat consumption, eating fast, consuming pickled vegetables, moldy food intake, preference for salty and acrid food along with esophageal lesion, HP infection, passive smoking, and a family history of cancer are risk factors for ESCC in Huaian area, China. Regular cleaning of food storage utensils, green tea consumption, and alcohol abstinence are important protective factors for ESCC in this high risk population. In addition, there was a significant association between the *GSTT1 *null genotype and risk of ESCC in male subjects. One potential limitation of the current study is the relatively smaller sample size, which may reduce statistical power for detecting gene-gene and gene-environmental differences. Hence, further studies with larger sample size and prospective cohort are mandated to confirm the present findings.

## Abbreviations

XME = xenobiotic-metabolizing enzymes; ESCC = esophageal squamous cell carcinoma; CYP = cytochrome P450; GST = glutathione S-transferase; EPHX = microsomal epoxide hydrolase; PCR = polymerase chain reaction; RFLP = restriction fragment length polymorphism; OR = odds ratio; CI = confidence interval; HP = helicobacter pylori.

## Competing interests

The author(s) declare that they have no competing interests.

## Authors' contributions

ZW carried out the genotyping studies, participated in the statistical analysis of the data and drafted the manuscript. LT participated in the design of the study, helped to coordinate sample usage and in drafting the manuscript. GS participated in the design of the study and in the acquisition of the epidemiological data and the samples. YT participated in genomic DNA extraction. YX participated in the acquisition of the samples. SW participated in the acquisition of the samples. XH organized the epidemiological field study and participated in the acquisition of the samples. WG participated in the statistical analysis of the data and helped to draft the manuscript. SBC participated in the statistical analysis of the data and helped to draft the manuscript. J-SW conceived of the study, participated in its design, and helped to draft the manuscript. All authors read and approved the final manuscript.

## Pre-publication history

The pre-publication history for this paper can be accessed here:



## Figures and Tables

**Table 1:** Characteristics of selected genes in this study

Loci	Gene location	Genotypes	Restriction enzymes	Methods	References
*CYP1A1*	3'-flanking	*6235 T to C*	*Msp*I	PCR-RFLP	Hong et al, 1998 [25]
*CYP1A1*	exon 7	*Ile462Val*	*Noc*I	PCR-RFLP	London et al, 2000 [27]
*CYP1B1*	exon 3	*Val432Leu*	*Eco57*I	PCR-RFLP	Saintot et al, 2004 [28]
*CYP2A6*	exon7–9	Deletion	-	Two step-PCR	Tan et al, 2001 [30]
*CYP2E1*	5'-flanking	*c1/c2*	*Rsa*I	PCR-RFLP	Tan et al, 2000 [22]
*GSTM1*	Whole gene	*GSTM1(-)*^1^	*No amplification*	Multiplex PCR	Wang et al, 2003 [23]
*GSTT1*	Whole gene	*GSTT1(-)*^1^	*No amplification*	Multiplex PCR	Wang et al, 2003 [23]
*GSTP1*	exon 5	*Ile105Val*	*Alw26*I	PCR-RFLP	Harries et al, 1997 [26]
*EHPX 113*	exon 3	*Tyr113His*	*Tth111*I	PCR-RFLP	de Jong et al, 2003 [29]
*EPHX 139*	exon 4	*His139Arg*	*Rsa*I	PCR-RFLP	de Jong et al, 2003 [29]

^1^null genotype.

**Table 2:** Demographic characteristics of the case and control populations

	Case n (%)	Control n (%)	OR(95%CI)	P
Male	60	60		
Female	47	47		
Mean age (yr)	62.9 ± 8.19	62.4 ± 8.35		
Smoking status				
None-smoker	40(37.4)	43(40.2)	1.00	
Smoker	67(62.6)	64(59.8)	1.14(0.64–2.04)	0.67
Passive smoking				
No	37(34.6)	54(50.5)	1.00	
yes	70(65.4)	53(49.5)	2.04(1.14–3.70)	0.02
Drinking status				
None-drinker	60(56.1)	58(54.2)	1.00	
Drinker	47(43.9)	49(45.8)	1.06(0.77–1.43)	0.70
Income (Yuan, Mean ± SD)				
Total	486.4 ± 425.80	592.0 ± 590.63	1.0(0.99~1.0)	0.10
Average	127.2 ± 98.68	148.2 ± 122.28	0.998(0.995~1)	0.15
Family history of cancer				
Negative	39(36.4)	49(45.8)	1.00	
Positive	68(63.6)	58(54.2)	2.90(1.41–5.95)	<0.01

**Table 3:** Univariate conditional logistic regression analyses of dietary and environmental factors and ESCC

Variable	OR (95%CI)	P
Personality	1.89(1.07~3.34)	0.03
Esophageal lesion	4.5(0.97–20.83)	0.05
HP infection	2.00(1.10~3.64)	0.02
Eating fast	3.25(1.70~6.21)	<0.01
Favor salty food	2.00(1.05~3.80)	0.03
Favor acrid food	2.08(1.07~4.03)	0.03
Fatty meat	3.00(1.19–7.56)	0.02
Pickled vegetables	2.55(1.27–5.11)	<0.01
Moldy food intake	2.27(1.12–4.61)	0.02
Clean up of storage utensils	0.27(0.12–0.62)	<0.01
Green tea consumption	0.25(0.09~0.67)	<0.01
Alcohol abstinence	0.13(0.03~0.54)	<0.01

**Table 4:** Multivariable conditional logistic regression analyses of risk factors for ESCC

Variable	OR (95%CI)	P
Esophageal lesion	11.63(1.13–119.33)	0.04
Eating fast	3.39(1.15–9.95)	0.03
Utensil clean up	0.11(0.02–0.49)	<0.01
Green tea consumption	0.13(0.03–0.62)	0.01
Family history of cancer	3.83(1.13–12.97)	0.03
HP infection	3.19(1.11–9.15)	0.03
